# Supplementary material for: Comparative Analysis of the GATA Transcription Factors in Five Solanaceae Species and Their Responses to Salt Stress in Wolfberry (Lycium barbarum L.)
Source: Genes (Basel). 2023 Oct 15;14(10):1943. doi: 10.3390/genes14101943 (PMC10606309; doi:10.3390/genes14101943)
Supplement: Supplementary file 1 [file genes-14-01943-s001.zip › Figure Supplementary/Figure S2 Phylogenetic relationship, conserved protein motifs, and gene structure in CaGATA genes.pdf]

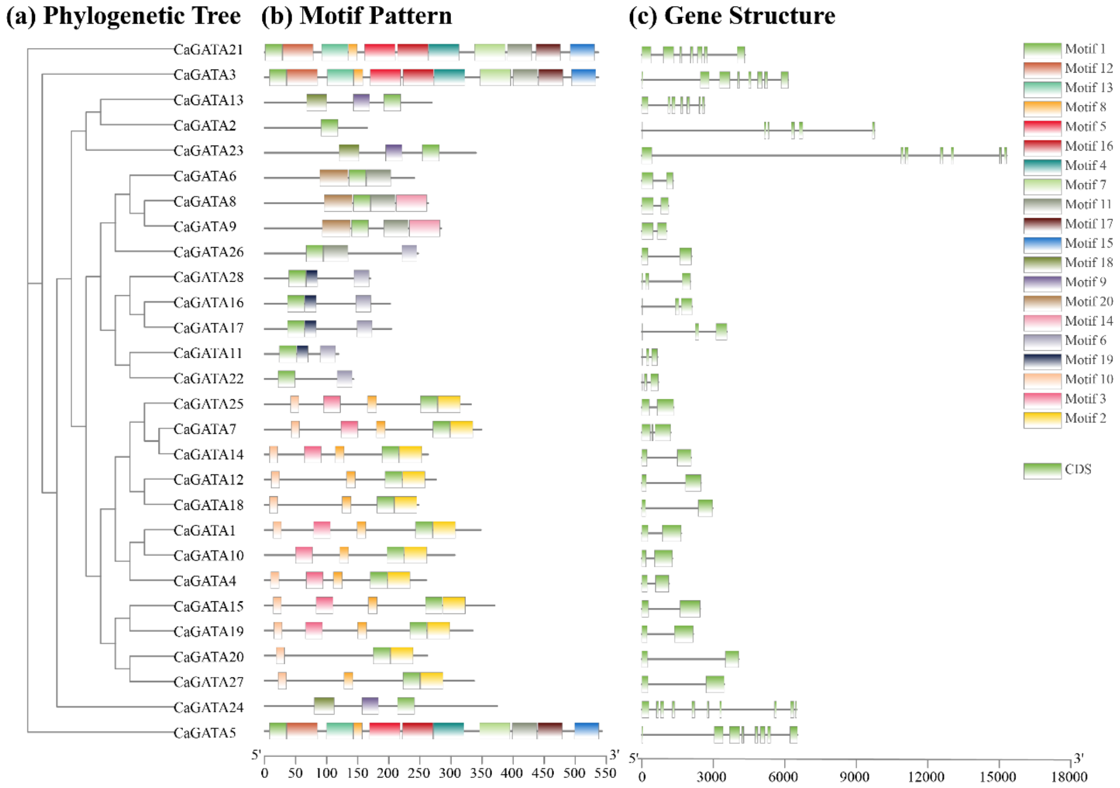

Figure S2. Phylogenetic relationship, conserved protein motifs, and gene structure in *CaGATA* genes. (a). The maximum-likelihood (ML) tree based on the full-length protein sequences of 28 CaGATA with 1000 bootstrap replicates. (b). The motif composition of CaGATA proteins. Twenty conserved motifs were performed using MEME, displayed in different colored boxes. The length of protein can be estimated using the scale at the bottom. (c). Exon/ intron structures of *CaGATA* genes. Green boxes indicate exons, and introns are represented with gray lines. The length of exons can be inferred by the scale at the bottom.
