# Supplementary material for: Comparative Analysis of the GATA Transcription Factors in Five Solanaceae Species and Their Responses to Salt Stress in Wolfberry (Lycium barbarum L.)
Source: Genes (Basel). 2023 Oct 15;14(10):1943. doi: 10.3390/genes14101943 (PMC10606309; doi:10.3390/genes14101943)
Supplement: Supplementary file 1 [file genes-14-01943-s001.zip › Figure Supplementary/Figure S5 Heat map of the correlations between biological replicates.pdf]

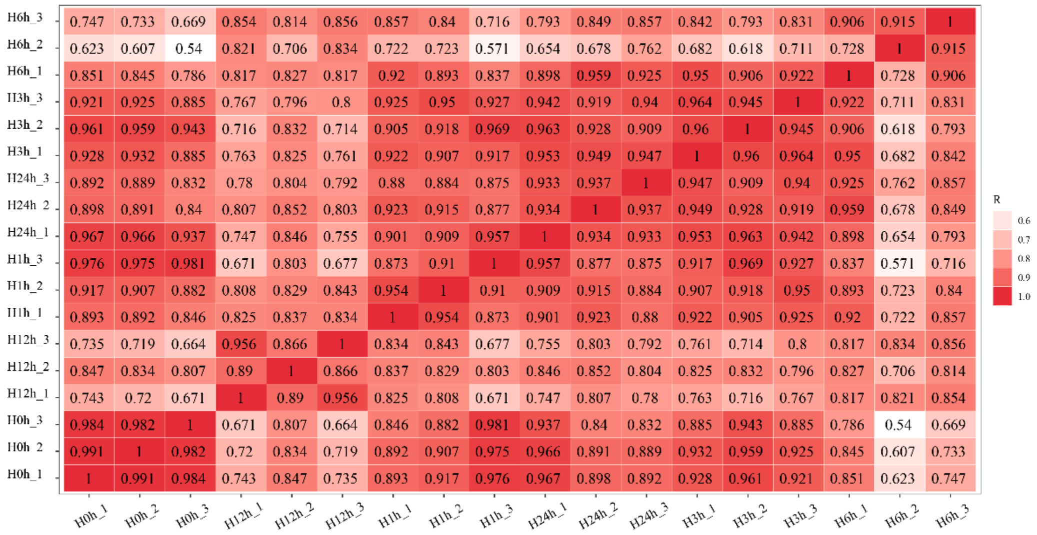

Figure S5 Heat map of the correlations between biological replicates. The PCC (Pearson correlation coefficient) values are quantitative indicators of relative expression levels of all genes in each sample.
