# Supplementary figures and images for: Comparative Analysis of the GATA Transcription Factors in Five Solanaceae Species and Their Responses to Salt Stress in Wolfberry (Lycium barbarum L.)
Source: Genes (Basel). 2023 Oct 15;14(10):1943. doi: 10.3390/genes14101943 (PMC10606309; doi:10.3390/genes14101943)

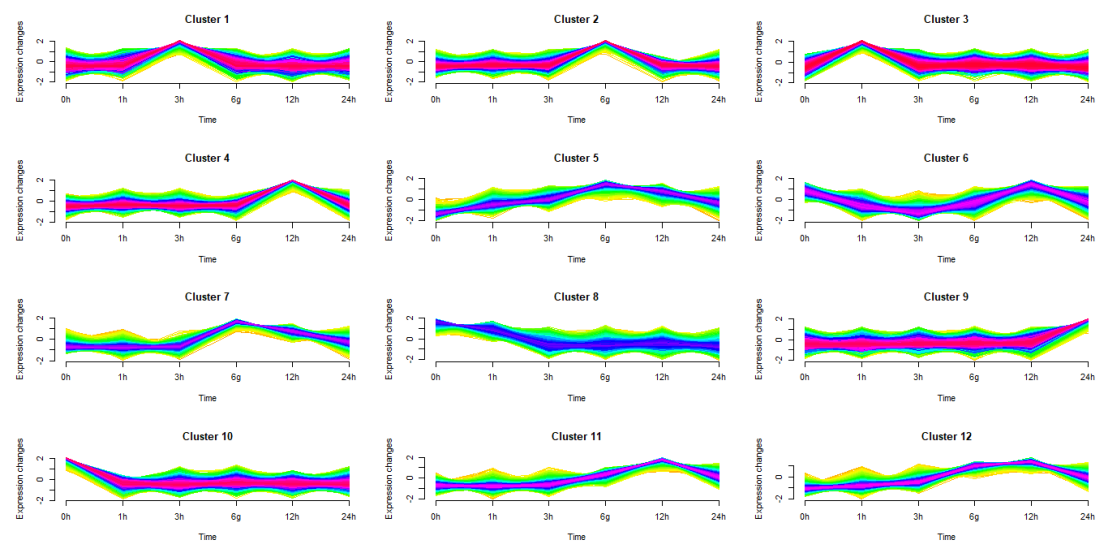

**Figure S6** Clustered gene expression profiles under salt stress treatment.

Supplement: Supplementary file 1 [file genes-14-01943-s001.zip › Figure Supplementary/Figure S6 Clustered gene expression profiles under salt stress treatment.pdf]
